# Characterization of Hyaluronic Acid-Coated PLGA Nanoparticles by Surface-Enhanced Raman Spectroscopy

**DOI:** 10.3390/ijms24010601

**Published:** 2022-12-29

**Authors:** Giuseppe La Verde, Antonio Sasso, Giulia Rusciano, Angela Capaccio, Sabato Fusco, Laura Mayol, Marco Biondi, Teresa Silvestri, Paolo A. Netti, Marco La Commara, Valeria Panzetta, Mariagabriella Pugliese

**Affiliations:** 1 Department of Pharmacy, University of Naples Federico II, Via Domenico Montesano, 80131 Naples, Italy; 2 Department of Physics “E. Pancini”, University of Naples Federico II, Via Cinthia, 80126 Naples, Italy; 3 Department of Medicine and Health Sciences “V. Tiberio”, University of Molise, Via Cesare Gazzani, 86100 Campobasso, Italy; 4 Department of Advanced Biomedical Sciences, University of Naples Federico II, Via Pansini, 80131 Naples, Italy; 5 Laboratory of Nanotechnology for Precision Medicine, Italian Institute of Technology, Via Morego, 16163 Genoa, Italy; 6 Department of Chemical, Materials and Production Engineering, University of Naples Federico II, Piazzale Vincenzo Tecchio, 80125 Naples, Italy; 7 Interdisciplinary Research Centre on Biomaterials CRIB, University of Naples Federico II, Piazzale Vincenzo Tecchio, 80125 Naples, Italy; 8 Center for Advanced Biomaterials for Healthcare @CRIB, Italian Institute of Technology, Largo Barsanti e Matteucci 53, 80125 Naples, Italy

**Keywords:** nanoparticles, hyaluronic acid, Raman spectroscopy, SERS, stability characterization

## Abstract

Nanoparticles (NPs) coated with hyaluronic acid (HA) seem to be increasingly promising for targeted therapy due to HA chemical versatility, which allows them to bind drugs of different natures, and their affinity with the transmembrane receptor CD-44, overexpressed in tumor cells. However, an essential aspect for clinical use of NPs is formulation stability over time. For these reasons, analytical techniques capable of characterizing their physico-chemical properties are needed. In this work, poly(lactide-co-glycolide) (PLGA) NPs with an average diameter of 100–150 nm, coated with a few 10 s of nm of HA, were synthesized. For stability characterization, two complementary investigative techniques were used: Dynamic Light Scattering (DLS) and Surface-Enhanced Raman Scattering (SERS) spectroscopy. The first technique provided information on size, polidispersity index, and zeta-potential, and the second provided a deeper insight on the NP surface chemicals, allowing distinguishing of HA-coated NPs from uncoated ones. Furthermore, in order to estimate formulation stability over time, NPs were measured and monitored for two weeks. SERS results showed a progressive decrease in the signal associated with HA, which, however, is not detectable by the DLS measurements.

## 1. Introduction

Traditional chemotherapy treatments, while being the gold standard in cancer treatment, present challenges for patient effects, such as inhibition of fast-turnover healthy cells (hair follicles, bone marrow, and gastrointestinal cells) [1], and multidrug resistance (MDR), typical of the most aggressive tumors [2]. To mitigate these adverse effects, in recent years, alternative approaches, usually going by the name of nanomedicine, have been developing.

This branch of medicine is producing promising results in diagnosis, imaging, drug administration, and therapy practices [3]. The strategy adopted involves the use of nanoparticles (NPs), of various natures, with diameters 1–100 nm [4]. NPs have, in fact, the potential to improve pharmacokinetics, stability, solubility, half-life, and accumulation in the target sites of chemotherapeutic drugs and, therefore, to enhance their therapeutic index compared to conventional therapy [5].

Nowadays, nanomedicine is starting to propose the use of NPs in combination with other cancer therapies, such as immunotherapy, hyperthermia, and radiotherapy. For example, NPs have been used as radiosensitizers which increase radiation damage to tumors [6] or, vice versa, the effects of ionizing radiation on cancer cell morphology and on the expression of surface-adhesion molecules [7,8] have been exploited to promote the cellular internalization of NPs [9,10].

Particle size, along with surface composition, plays a crucial role for drug delivery. NPs with dimensions below 200 nm are not detected by the mononuclear phagocytic system, and therefore they can remain in circulation longer [11], but at the same time, they can be directly eliminated by the renal system. Clearly, by optimizing the carrier size it is possible to promote passive penetration, for example by enhanced permeability and retention (EPR) effect [12]. In addition, NP functionalization can exploit active recognition mechanisms or can allow the crossing of a low-permeability barrier, such as the blood–brain barrier) transporting the drug to a target organ [13].

Among the molecules used to promote NP uptake, hyaluronic acid (HA) plays a major role for its tropism toward the cluster determinant 44 (CD-44), which is overexpressed in a wide array of malignant tumors [14,15,16], and is therefore attractive for the formulation of NPs for active tumor targeting [17,18].

In this *scenario*, it is crucial to prepare a stable formulation that guarantees the safety and efficacy of the pharmaceutical compounds. Critical points involve all steps of the process from synthesis to transport and storage. Although both physical and chemical alteration processes are known, ensuring the stability of a formulation remains a complex problem that is not yet easy to manage [19].

The aim of this work is to prepare HA-coated NPs and investigate their surface characteristics by combining complementary techniques. In particular, NPs were produced through the nanoprecipitation technique [20] using HA with a mean molecular weight of 800 kDa, which maximizes NP internalization in tumor cells [21]. To study the formulation stability, two different techniques were used: Dynamic Light Scattering (DLS) and Surface-Enhanced Raman Scattering (SERS) spectroscopy. DLS represents one of the most used techniques to monitor physico-chemical parameters of NPs; nevertheless, it does not provide information on the chemical nature of possible variation in these parameters. For example, for HA-coated NPs, the increase in mean diameter over time remains difficult to interpret; it could be ascribed to both the alteration of the tertiary structure of the HA or to the aggregation of HA debris in the solution [22,23,24].

Raman Spectroscopy (RS) is a well-established label-free technique with high chemical specificity (finger-printing character) [25]. However, being based on inelastic scattering, its cross-section is rather low (10–30 cm^2^), limiting its application to cases where high sensitivity is not required. This drawback has been overcome in recent decades by a variant of it, Surface-Enhanced Raman Spectroscopy (SERS), based on localized surface plasmon resonances (LSPR) which occur in metallic nanostructures (mainly Au and Ag) irradiated by laser radiation of a suitable wavelength. In particular, a huge amplification of the laser field (of typically between six and eight orders of magnitude) originates in the gaps which are formed in these nanostructures (hot-spots). This allows the detection of analytes at very low concentrations, down to the single-molecule regime. In addition, since the SERS amplification decreases rapidly (within a few tens of nm) moving away from the metal surface, the technique is particularly suitable for the study of interfaces, since the bulk contribution becomes negligible with respect to the interface placed in the proximity of the SERS substrate.

The SERS technique has been used in a very large number of applications in the fields of materials, chemistry, and biology [26,27,28,29]. For example, our group has analyzed the spore coat formation in Bacillus subtilis using both SERS [30] and Tip-Enhanced Raman Scattering (TERS) techniques [31]. However, according to our knowledge, few studies have been dedicated to the characterization of composite NPs and, in particular, to their external surface.

The novelty proposed in this work is to study the structure of HA-coated NPs composed by a core and a thin outer layer. SERS is particularly suitable for this type of investigation because it combines the high sensitivity required to detect and discriminate the external thin HA layer from the rest of the NP. More specifically, we demonstrate that the SERS technique allows distinguishing of HA-coated from uncoated NPs. In addition, by following over the time the evolution of the signal intensity corresponding to HA, we also studied HA-coating degeneration (aging). This approach holds promise for a reliable characterization of the external shell of newly developed NPs for biomedical and biotechnological applications.

## 2. Results and Discussion

### 2.1. DLS Measurements

Two different types of formulations were prepared by nanoprecipitation technique: control NPs, consisting of an organic blend of poly (lactide-co-glycolide, PLGA) and poloxamers, (F68 and F127) and HA-decorated NPs, hereinafter indicated as PP-NPs and HA-PP-NPs, respectively.

In order to evaluate the stability over time of PP-NPs and HA-PP-NPs stored at 4 °C DLS measurements were carried out at room temperature and at three different time points, 0, 7, and 14 days (T_0_, T_7_, T_14_). Mean diameter, PDI, and ζ-potential found for these times are reported in Table 1.

At the first time point (T_0_), PP-NPs exhibited a mean diameter of about 100 nm and negative ζ-potential of −26 mV. In the case of HA-PP-NPs, the significant enhancement of their mean diameter of about 30% (Kruskal–Wallis test, *p* < 0.001) and the significant reduction in their ζ-potential up to −34 mV (Kruskal–Wallis test, *p* < 0.01) indicate a successful external decoration with HA [32].

Dimensional stability was established by evaluating the hydrodynamic diameter in water, up to T_14_. Both NPs were stable up to T_14_ and no phenomenon of self-aggregation was observed.

On the contrary, the enhancement of ζ-potential for HA-PP-NP formulation already at T_7_ (Kruskal–Wallis test, *p* < 0.001) and for PP-NPs at T_14_ (Kruskal–Wallis test, *p* < 0.005) indicated a likely alteration of the NP surface that could be ascribed to poloxamers and HA (the two layers most exposed and therefore susceptible to changes in PP-NPs and HA-PP-NPs, respectively). It must also be specified that ζ-potential changes in the time frame of the observation can be reasonably ascribed to statistical fluctuations in the measurement.

To obtain direct information on the physico-chemical structure of the NPs, a spectroscopic technique was employed to monitor the evolution of the external regions of NP.

### 2.2. Spontaneous Raman Spectroscopy of Bulk Materials

Preliminary SR measurements were carried out on the single components forming NPs, i.e., PLGA, F127, F68, and HA, in order to know their main spectroscopic features.

**Figure 1:**
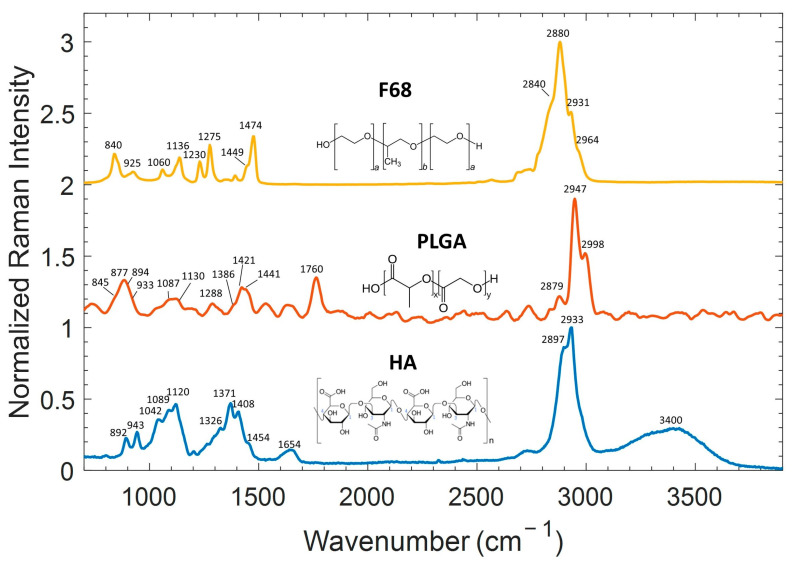
Comparison of Raman spectra of (yellow) F68 (a = 76, b = 29), (red) PLGA (x = 50, y = 50), and (blue) HA powders acquired with an integration time of 50 s and a Raman probe of 15 mW. For a better comparison, spectra were normalized and shifted.

Measurements of all these chemical species were performed using samples in the form of powder. Figure 1 shows the averaged Raman spectra of the main components in the 300–3900 cm^−1^ spectral range, acquired with a laser power of 15 mW and an integration time of 50 s. Since the Raman spectrum of F127 is quite similar to F68, as will be explained later, for sake of simplicity it is not plotted herein.

Following the literature concerning HA Raman studies [22,33,34,35], the main bands could be assigned. The relatively narrow bands at ~892 cm^−1^ and ~943 cm^−1^ are ascribed to β-linkages and C-C stretching modes, while the broad bands around 1000 cm^−1^ derive from the overlapping of the C-C and C-O stretching modes (~1042 cm^−1^), C-OH bending mode of an acetyl group (~1089 cm^−1^), and C-OH and C-H bending modes (~1120 cm^−1^). The other wide band around 1400 cm^−1^ also results from the superposition of several vibrational modes: an amide III band at ~1326 cm^−1^, and asymmetric and symmetric CH3 bending vibrational modes at ~1371 cm^−1^ and ~1454 cm^−1^, respectively. The bands at ~1408 cm^−1^ result from CH deformation and the symmetric COO− stretching mode which is a vibration sensitive to hydrogen bonding, while the band at ~1654 cm^−1^ is ascribed to C=C and amide I C=O vibrations, with low-frequency shoulders deriving from asymmetric COO- stretching vibrational modes. Finally, the bands at 2897 and 2933 cm^−1^ correspond to the C-H and NH stretching modes. In addition, the band around 3400 cm^−1^ is ascribed to the OH-free mode and to symmetrical and asymmetrical OH stretching modes.

In the case of PLGA, based on previous works [36,37], the peaks detected are ascribable to vibrational modes of lactic (LA) or/and glycolic (GA) units. PLGA is a copolymer formed by the two units: poly(d,l-lactide) (PLA) and poly(glycolide) (PGA) polymers. As a matter of fact, the spectra of PLGA shows some differences from the spectra of single units due of the interaction of the two polymers. In particular, the main interesting features are the 1760 cm^−1^ band assigned to C=O stretching of the ester groups and the stretching modes of the CH_3_ (LA) and CH_2_ (GA) groups at 2998 and 2947 cm^−1^, respectively. 

Regarding the Raman spectrum of F68 [38], the assignments of the main vibrational modes are listed in Table 2. The F68 is a poloxamer, i.e., a triblock copolymer composed of a central hydrophobic chain of polyoxypropylene (PPO) linked to two hydrophilic chains of polyoxyethylene (PEO). The relative intensities of several peaks in Raman spectrum are dependent on the PPO/PEO ratio and the conformation of the copolymer. In this case, both F68 and F127 are characterized by a low ratio of PPO/PEO, corresponding to 0.19 and 0.32, respectively. For this reason, Raman spectra are mostly due to PEO unit contribution, which gives rise to the most prominent bands centered at 840 cm^−1^ (ρ(CH_2_)), 1136 cm^−1^ (ν(CH_2_), ν(CC)), 1275 cm^−1^ (δ(CH)), 1474 cm^−1^ (CH_2_ scissoring), and 2880 cm^−1^ (ν(CH_2_)) [39].

From the comparison of the Raman spectra of the three components, HA, PLGA, and F68, shown in Figure 1, a certain overlap between the bands is highlighted, especially for the vibrations involving CH_n_.

The simultaneous presence of these three components, as occurs in HA-PP-NPs, makes spectra analysis difficult, as will be discussed later.

### 2.3. SERS and Raman Analysis of HA

**Figure 2:**
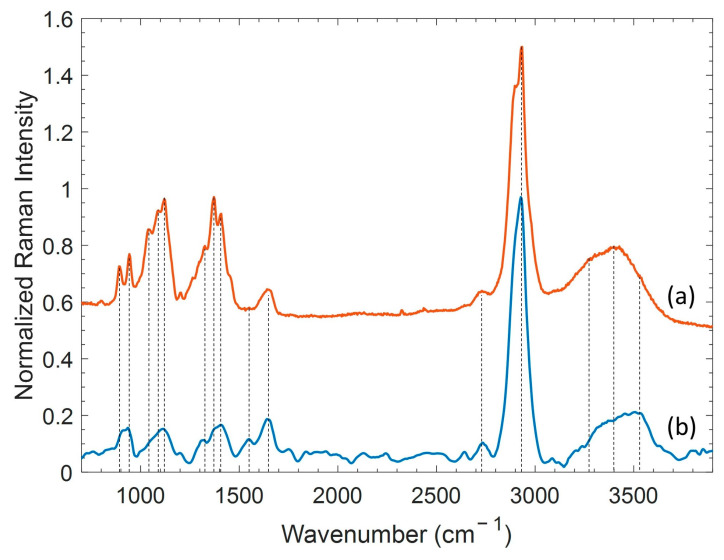
Comparison of Raman spectrum of HA powder (a) and SERS spectrum of HA solution (b). The spectra were normalized and shifted.

As already mentioned, the SERS technique allows the detection of chemical species at low concentration, preserving the fingerprinting character of Raman spectroscopy. In Figure 2, the SR of a solid HA sample, discussed in the previous paragraph, and the average SERS spectrum of HA solution at the concentration of 2.5 mg/mL are compared.

The SERS measurements were performed by placing a volume of 20 µL of solution upon the SERS substrate, allowing solvent evaporation. The evaporated droplet left on the surface a spot with a diameter of about 5 mm. After that, a map of 100 points (spectra) in an area of 25 × 25 µm^2^ was collected four times, in four corresponding different points of the dried droplet (*p* = 300 µW, τ = 1 s). By averaging the acquired SERS spectra, possible SERS intensity variability due to the inhomogeneous distribution of molecules on the surface (coffee-ring effect [40]) was minimized.

By comparing the Raman and SERS spectra reported in Figure 2, it can be appreciated that both the bandwidth and the relative intensity of the bands show some differences. The broadening of the peaks reflects the order character of the sample: the narrower the peak, the more ordered the structure is. Thus, the SERS spectrum of HA dissolved in solution shows broader bands, probably because of the random orientation of the molecules over the metal surface. In addition, due to the surface selection rule for SERS [41], vibrational modes can be amplified or depressed according to if polarizability changes normally or parallel to the surface.

### 2.4. SERS of PP-NPs and HA-PP-NPs

**Figure 3:**
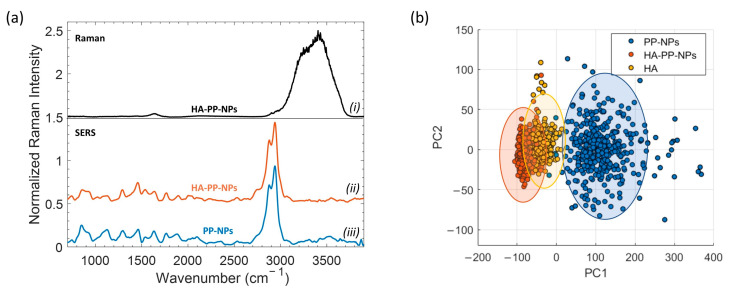
(**a**) Comparison of (i) Raman and SERS spectra of HA-PP-NP (ii) and PP-NP (iii) samples. The spectra were normalized and shifted; (**b**) score plot of PC1 versus PC2 for PP-NPs, HA-PP-NPs, and HA.

We analyzed HA-PP-NPs samples at a density of about 8 × 10^11^ particles/mL, estimated from DLS measurements. Therefore, by using SR this means that in the detection volume V_det_ = π w^2^ × h_eff_ = 2.3 µm^2^ are present in average ~2 particles, a too-low number of particles compared to the sensitivity of SR. Figure 3a, trace (i) shows the spectrum of HA solution acquired by SR with a power of 30 mW and a time integration of 50 s.

Except for a weak signal detectable in the region around ~2900 cm^−1^, the only observed bands correspond to the bending and stretching mode of the water molecule at 1640 and 3400 cm^−1^, respectively. This proves that SR fails at the considered concentration. The Raman response is different when SERS is used: Figure 3a, traces (ii) and (iii), report the average SERS spectrum of HA-coated and uncoated NPs acquired after the evaporation of a 20 µL droplet deposited upon a SERS substrate. It is also worth noting that the power used for SERS measurements was particularly low (P = 145 µW) and the exposure time quite short (τ = 2 s) in order to avoid possible photo-thermal damage.

A careful comparison between the two SERS spectra shows significant differences. These mainly concern the bands around 850 cm^−1^ and 2750 cm^−1^. These differences are better highlighted through the use of the principal component analysis (PCA) performed on the band at 2750 cm^−1^ (see Figure 3b). For this analysis, we considered a number N = 400 SERS spectra for each sample; a set of HA SERS spectra was also included as control. SERS spectra in the PCA space were projected according to the first two principal components. The score plot shows a clear difference between the two populations (PP-NPs and HA-PP-NPs) along PC1. Notably, the HA-PP-NP population tends to overlap with the set of spectra of HA. This result is consistent with the fact that HA-coated NPs expose this polymer to the SERS substrate and, hence, a certain similarity is expected between these two populations.

### 2.5. HA-PP-NP Aging SERS Analysis

**Figure 4:**
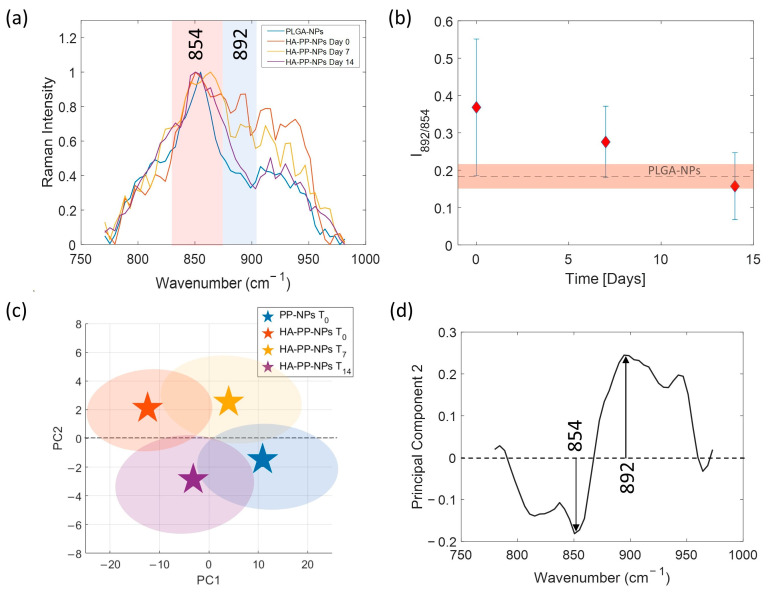
(**a**) Comparison of SERS spectra relative to PP-NPs T_0_ and HA-PP-NPs T_0_, T_7_, T_14_; (**b**) Plot of the ratio intensities I_892/854_ as a function of time. The ratio value relative to PP-NP T_0_ spectrum is reported as a dashed horizontal line and the corresponding error bar as a red box; (**c**) Score plot of PC1 versus PC2 for PP-NPs T_0_ and HA-PP-NPs T_0_, T_7_, T_14_; (**d**) Loading plot of PC2.

In the previous section, we established that HA-PP-NPs can be chemically distinguished from PP-NPs by observing differences in their SERS spectra. Since HA is not covalently grafted to the NPs, it is to be expected that the stability of the particles is not durable over time. In fact, HA can be subjected to various processes that modify its consistency: it can simply be released and dispersed in the solution, or it can undergo a molecular rearrangement, such as swelling. In both cases, this can lead to an alteration of the Raman bands in terms of their intensities or frequency shifts. To investigate aging effects on HA-PP-NPs, SERS and DLS measurements were carried out in parallel and we adopted the following procedure. Once the HA-PP-NP sample was produced, we carried out a measurement campaign by taking a small aliquot of material from the mother solution, which was kept at a temperature of 4 °C. SERS measurements were performed depositing a droplet of volume 20 µL on the SERS substrate, allowing it to dry in ambient air. For each sample, a series of 400 spectra was acquired over randomly selected points of the substrate. This protocol was applied to fresh sample (initial time T_0_) and repeated after one week (T_7_) and two weeks (T_14_). We observed macroscopic differences by comparing the average SERS spectra obtained at times, T_0_, T_7_, and T_14_ in the spectral region between 750 and 1000 cm^−1^, as reported in Figure 4a. For complete comprehension, the average SERS spectrum of PP-NPs at T_0_ was considered as reference. Following the Raman assignments of the HA spectrum discussed previously, the peak centered at 892 cm^−1^ is clearly ascribed to the presence of HA on the surface of coated PP-NPs. Differently, the band at 854 cm^−1^ is of no immediate assignment because it could be related to both PLGA and F68. Hence, more in general, this band could represent the contribution of the inner part of the NPs. In Figure 4a, the spectra have been normalized to the signal at 854 cm^−1^. A convenient way of showing this variation is reporting the ratio of the intensities of the two peaks, I_892/854_, as a function of time (see Figure 4b). As we can see, I_892/854_ decreases by reaching approximately the value corresponding to PP-NPs. The fact that the behavior of HA-PP-NPs tends to that of naked PP-NPs suggests that HA is released over time, rather than subjected to some molecular reorganization with the rest of the nanoparticle core.

This claim is further confirmed by analyzing the four populations of SERS spectra, corresponding to the above-mentioned samples, with the PCA in the more significative spectral range 750–1000 cm^−1^. Figure 4c reports the outcomes of this analysis by showing the resulting cluster centroids of each group in the 2-D PCA score plot. At first glance, a net separation of the clusters distributed in PC space can be observed both along PC1 and PC2. Intriguingly, a clear interpretation of the differentiation along the second PC can be revealed by looking at the corresponding loading plot of PC2 reported in Figure 4d. The analysis of the loadings highlights an important inverse correlation between the peaks centered at 854 and 892 cm^−1^. The positive loading indicates that the groups lying along the positive direction of PC2, i.e., HA-PP-NPs at T_0_ and at T_7_, are characterized by the presence in their SERS spectra of the spectral line centered at 892 cm^−1^, which is equivalent to the expression of the presence of the HA in these samples. On the contrary, the populations relative to PP-NPs at T_0_ and HA-PP-NPs at T_14_, grouped along the negative direction of PC2, are characterized by spectral content of the peak at 854 cm^−1^ which has a negative loading. This result reflects exactly the tendency observed in Figure 4b in which the intensity of the peak relative to 892 cm^−1^ of HA-PP-NPs reduces, approaching finally at T_14_ the same value of PP-NPs at T_0_ spectrum.

## 3. Materials and Methods

### 3.1. NP Formulation

Poly (lactic-co-glycolic acid) (PLGA), lactide:glycolide 50:50, was obtained from Sigma-Aldrich (St. Louis, MO USA), while poloxamers (PEOa-PPOb-PEOa F127 (a = 100 and b = 65) and F68 (a = 76 and b = 29) were from Lutrol Lutrol Basf, Ludwigshafen, Germany. HA with a weight-average MW of 830 kDa was kindly provided by Altergon, Avellino, Italia.

**Figure 5:**
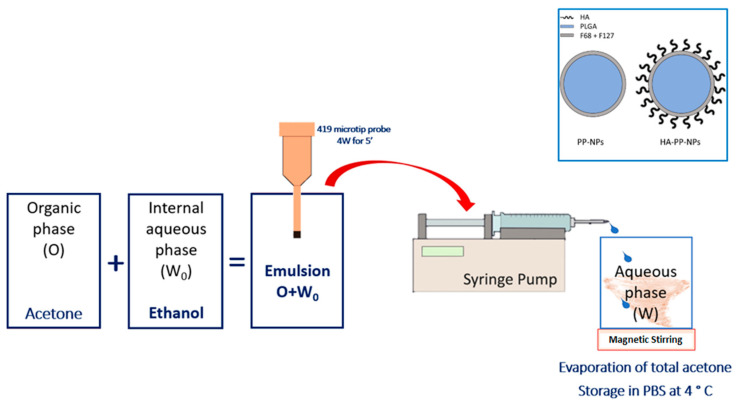
Schematization of NP formulation process with the nanoprecipitation technique. The inset in the upper right corner shows the two possible formulations.

Processes of NP formulation are schematized in Figure 5. NPs were prepared using the nanoprecipitation technique followed by solvent evaporation, as previously described [42]. Briefly, for both formulations (PP-NPs and HA-PP-NPs) an organic phase (O) made up of PLGA, F68, and F127 (mass ratio 1:0.5:0.5; overall polymer concentration was 1.5% *w/v*) in 5 mL of acetone was prepared. Subsequently, 640 µL of ethanol (internal aqueous phase, W_0_) was added and W_0_/O emulsion was obtained by sonication (3000 sonicator, Misonix, Farmingdale, NY, USA; 5 min, 4W, 419 microtip probe). The emulsion was added dropwise, through a syringe pump at a volumetric flow rate of 333.3 μL/ min, to 40 ml of an external aqueous phase (W) containing F68 and F127 (0.0375 *w/v* each). In the case of HA-PP-NPs, 30 mg of HA 830 kDa was added to the W phase. Subsequently, acetone was evaporated for about 4 h under magnetic stirring. The resulting NP suspension was divided into 2 mL tubes and centrifuged three times (Hettich Zentrifugen, Tuttlingen Germany; 10,000 rpm, 10 min). After the first two centrifuges, 1 mL of supernatant was discarded and replaced with an equal volume of double-distilled water. After the last centrifuge, 1.5 mL of supernatant was removed, and 0.5 mL of PBS (sodium chloride, potassium chloride, and disodium phosphate) was added. The obtained formulation was filtered with 0.2 μm filters and stored at 4 °C.

### 3.2. Dynamic Light Scattering

Dynamic Light Scattering (DLS) is the most used technique to analyze particles at nanoscale. The DLS technique is based on scattering due to a laser beam that strikes a colloidal suspension. Therefore, the size of the particles can be extrapolated from analysis of the fluctuations in the intensity of the scattered light using the Stokes–Einstein equation:

D = (k_B_ T)/(6πηR_H_), (1)

where D is the translational diffusion coefficient, kB is the Boltzmann constant, T is the absolute temperature, η is the viscosity coefficient of the solvent, and R_H_ is the hydrodynamic radius. The NP solutions were stored at 4 °C for 14 days. Then, mean diameter and PDI of NPs were obtained through DLS measurements by laser light scattering (Zetasizer Nano ZS, Malvern Instruments, Malvern, UK) at room temperature on NP suspensions in ultra-water at 0.1 mg/mL at three different time points (0, 7, and 14 days). Twelve runs were run for each sample.

In addition, through the electrophoretic mobility, the zeta-potential can also be estimated [43]. If the ζ value is high, electrostatic repulsion prevents the aggregation of the dispersed particles, while when it is low the attractive forces prevail, and the species give rise to coagulation phenomena. Negative values between −20 and −40 mV correspond to particles that are unlikely to aggregate, are stable, non-toxic, and have a high penetration capacity. Positive ζ values are associated with particles usually toxic and potentially fusogenic with cells [44,45].

### 3.3. Raman Analysis

Raman and SERS measurements were performed using a commercial system (WiTec, mod Alpha300 RA) equipped with a frequency-doubled Nd/YAG laser emitting at 532 nm a maximum power of 30 mW. The laser beam was focused with a 20× objective lens. Scattered photons were collected in a back-scattering geometry through the same focusing objective lens. Inelastic photons were separated from elastic photons using an edge-filter and sent via a 100 µm core fiber to a high-throughput spectrometer. Spectra were acquired with a thermoelectrically cooled CCD camera (T = −60 °C).

### 3.4. SERS Substrates

**Figure 6:**
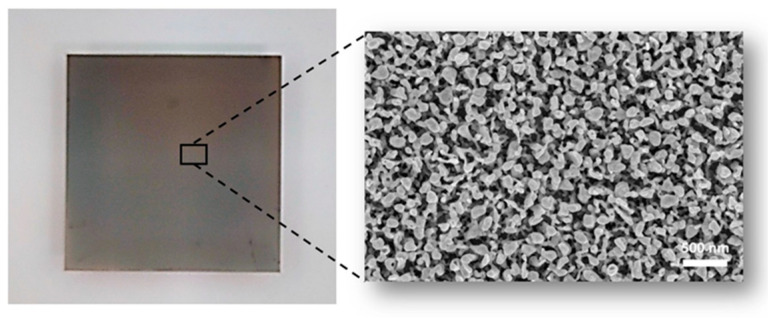
Digital photograph of SERS substrate fabricated on a 15 × 15 mm^2^ glass coverslip and SEM image of the nanostructured Ag film, exhibiting the typical coral-like morphology.

Details on the procedure used for the fabrication of SERS substrates are reported in [46]. Briefly, a Ag film of 30 nm was deposited on a Cr/Au bilayer (3 nm Cr, 10 nm Au), preliminarily deposited on a 15 × 15 mm^2^ glass coverslip in order to optimize the Ag adhesion. Depositions were performed using a magnetron sputtering system (Q300T D Plus, Quorum Tech., Lewes, England). The so-produced films were rather flat, with an average roughness of about 2 nm. In order to induce a nanostructuration of the Ag film (necessary to make the films SERS-active), a cold plasma treatment was applied to them for 90 s by using an inductively coupled plasma system (PDC-32G-2, Harrick Plasma, Ithaca, NY, USA) in a synthetic air atmosphere. This operation led to a coral-like porous nanostructured surface. Nevertheless, the presence of oxygen in the discharge, essential to sustain the nano-structuration mechanisms of the surface, creates a thin layer of Ag-oxide with consequent loss of plasmonic properties. The metallic character of the pristine surface, without altering its nanostructured pattern, was restored through a further 50 s of plasma treatment in an Ar atmosphere [47]. In Figure 6, an image of our substrate obtained with scanning electronic microscopy (SEM) is shown.

The SERS substrates so-produced were characterized using 4-MBA as a probe molecule. Interesting performances in terms of enhancement factor (average EF ∼10^7^) and spatial reproducibility were obtained.

### 3.5. Statistical Analysis of DLS

Descriptive statistics of DLS data (mean diameter, PDI, and ζ-potential) consisted of mean values and standard deviation. The normality of data was checked by the Shapiro–Wilk test (*p* values < 0.05 indicates non-normal distribution). Statistical comparisons were performed with a Student’s unpaired test when data exhibit a normal distribution. Otherwise, a nonparametric Kruskal–Wallis test was used. Differences were considered significant for *p* values less than 0.05.

### 3.6. Statistical Analysis of SERS Spectra

PCA is a well-known multivariate statistical tool suitable to analyze multidimensional datasets. Its basic principle consists of an orthogonal linear transformation of the initial dataset (SERS spectra in our case) into a new coordinate system such that the greatest variance in the projected data comes to lie on the new coordinates. The first coordinate, called the first principal component (PC1), explains the maximum variance in the dataset; the following components consider the residual variance, and so on. Each PC is composed of scores and loadings: the scores represent the variance on sample direction, hence being used to highlight similarities/dissimilarities between samples; the loadings represent the variance on wavenumber direction, being used to identify possible spectral features responsible for cluster differentiation. Therefore, PCA allows condensation of the score plot, the acquired spectra in a set of points having as coordinates the respective PCi, i = 1, 2, …, n. The affinity between spectra referring to different samples appears from the degree of clustering of the points in the PC space. In this experiment, SERS spectra were pre-treated by using a custom-made routine developed in order to eliminate spurious cosmic ray contributions and to subtract a fourth-order polynomial background contribution.

## 4. Conclusions

This work confirms the need to combine the more common DLS technique with the SERS technique to characterize the physico-chemical properties of HA-coated and uncoated NPs. In particular, the information obtainable from the DLS, such as dimensions, polydispersion index, and zeta-potential, are not able by themselves to fully describe the nature of the external coating of the NPs and their evolution over time. In this regard, the literature has not yet shed full light. In our study, the SERS technique was chosen for its high chemical specificity, its high sensitivity, and for the sub-diffraction resolution along the direction normal to the SERS substrate. All these features were precious for detecting the thin HA layer around the PP NPs, distinguishing it from the rest of the core NPs. This allowed us to clearly distinguish HA-coated from uncoated NPs. Even more interesting, SERS allows monitoring of the time evolution of the surface chemistry of the NPs, obtaining information about their stability. In this experiment, the DLS results show that, over two weeks, particle sizes do not have a significant tendency to change. On the other hand, zeta-potential unequivocally shows the tendency of NPs to be less charged. Similarly, by analyzing the SERS spectra, it is observed that, in the same time period, the intensity of the vibrational band 892 cm^−1^, associated with HA, tends to decrease compared to at 854 cm^−1^, probably corresponding to PLGA or to poloxamer.

These results offer further opportunities, for example, to optimize the NP synthesis procedure to make NPs as stable as possible over time and to integrate different analysis techniques to obtain more detailed information.

## Figures and Tables

**Table 1:** DLS measurements at T_0_, T_7_, and T_14_.

Formulation	Mean Diameter (nm)	PDI	ζ (mV)	Time
PP-NPs	109 ± 11	0.11 ± 0.04	−26 ± 6	T_0_
HA-PP-NPs	145 ± 16	0.14 ± 0.076	−34 ± 8	
PP-NPs	118 ± 15	0.13 ± 0.05	−26 ± 5	T_7_
HA-PP-NPs	135 ± 23	0.15 ± 0.05	−24 ± 4	
PP-NPs	117 ± 10	0.16 ± 0.05	−23 ± 2	T_14_
HA-PP-NPs	142 ± 17	0.13 ± 0.06	−29 ± 4	

**Table 2:** Raman assignments of F68, PLGA, and HA (ν = stretching, ρ = rocking, δ = bending, τ = twist).

F68	PLGA	HA	Tentative Assignment (cm^−1^)
840	845		ρCH_2_
	877		νC-COO
	894		ρCH_2_, νC-C
		892	β-linkages
925			ρCH_2_
	933		ρCH_2_, νC-C
		943	νC-C, νC-O-C
		1042	νC-C, νC-O
1060			ρCH_2_, νC-O
	1087	1089	δC-OH
		1120	δC-OH, δCH
	1130		ρCH_3_ asym
1136			νCH_2_, νC-C
1230			τCH_2_
1275	1288		δCH
		1326	Amide III
	1386	1371	δCH_3_ sym
		1408	δCH, δCOO-
	1421		δCH_2_
1449			CH_2_ scissor
	1441	1454	δCH_2_, δCH_3_ asym
1474			CH_2_ scissor
		1654	νC=C, Amide I C=O
	1760		νC=O
2840			νCH_2_ sym
2880	2879		νCH_2_ asym
		2897	νCH
		2933	νNH
2931			νCH_3_ sym
	2947		νCH_2_ sym
2964	2998		νCH_3_ asym
		3200/3500	νOH

## Data Availability

Data are contained within the article.
